# The trend in mental health-related mortality rates in Australia 1916-2004: implications for policy

**DOI:** 10.1186/1743-8462-7-3

**Published:** 2010-01-07

**Authors:** Darrel P Doessel, Ruth FG Williams, Harvey Whiteford

**Affiliations:** 1 Australian Institute for Suicide Research and Prevention, Griffith University, Mt Gravatt, Australia; 2 Queensland Centre for Mental Health Research, School of Population Health, The University of Queensland, Australia; 3 School of Economics and Finance, Victoria University, Sunbury Campus, Australia

## Abstract

**Background:**

This study determines the trend in mental health-related mortality (defined here as the aggregation of suicide and deaths coded as "mental/behavioural disorders"), and its relative numerical importance, and to argue that this has importance to policy-makers. Its results will have policy relevance because policy-makers have been predominantly concerned with cost-containment, but a re-appraisal of this issue is occurring, and the trade-off between health expenditures and valuable gains in longevity is being emphasised now. This study examines longevity gains from mental health-related interventions, or their absence, at the population level. The study sums mortality data for suicide and mental/behavioural disorders across the relevant ICD codes through time in Australia for the period 1916-2004. There are two measures applied to the mortality rates: the conventional age-standardised headcount; and the age-standardised Potential Years of Life Lost (PYLL), a measure of premature mortality. Mortality rates formed from these data are analysed via comparisons with mortality rates for All Causes, and with circulatory diseases, cancer and motor vehicle accidents, measured by both methods.

**Results:**

This study finds the temporal trend in mental health-related mortality rates (which reflects the longevity of people with mental illness) has worsened through time. There are no gains. This trend contrasts with the (known) gains in longevity from All Causes, and the gains from decreases achieved in previously rising mortality rates from circulatory diseases and motor vehicle accidents. Also, PYLL calculation shows mental health-related mortality is a proportionately greater cause of death compared with applying headcount metrics.

**Conclusions:**

There are several factors that could reverse this trend. First, improved access to interventions or therapies for mental disorders could decrease the mortality analysed here. Second, it is important also that new efficacious therapies for various mental disorders be developed. Furthermore, it is also important that suicide prevention strategies be implemented, particularly for at-risk groups. To bring the mental health sector into parity with many other parts of the health system will require knowledge of the causative factors that underlie mental disorders, which can, in turn, lead to efficacious therapies. As in any case of a knowledge deficit, what is needed are resources to address that knowledge gap. Conceiving the problem in this way, ie as a knowledge gap, indicates the crucial role of research and development activity. This term implies a concern, not simply with basic research, but also with applied research. It is commonplace in other sectors of the economy to emphasise the trichotomy of invention, innovation and diffusion of new products and processes. This three-fold conception is also relevant to addressing the knowledge gap in the mental health sector.

## Background

Burden of disease studies indicate that the impact of mental disorders is considerable [1-3], while the latest Australian Institute of Health and Welfare (AIHW) report on relative health expenditures by disease groups indicates that mental disorders are the seventh most expensive disease category in Australia [4,5]. Various dimensions of mortality associated with mental disorders are not trivial. There are some meta-analyses indicating the excess mortality associated with these disorders--both natural and unnatural causes increase the risk of premature death for mentally ill people [6,7]. Also, a single international meta-analysis, focussing only on suicide, shows a heightened suicide risk is associated with almost all mental disorders [8]. Another approach--that of the psychological autopsy--has found that about 90 per cent of people who die by suicide have at least one mental disorder at the time of death [9]. Several Australian studies that have examined various aspects of mortality from mental disorders are now available, their focus largely being on suicide [10-17].

The present study measures the time-trend in the mental health-related mortality rate. The term "mental health-related mortality" is defined as the sum of deaths from mental and behavioural disorders and suicide. Apart from the conventional headcount measure, some studies have applied an alternative measure, the potential years of life lost (PYLL) to suicide [18,19]. The PYLL metric originated in the 1940s for the evaluation of tuberculosis prevention programs, when it had become apparent that headcount (only) measurement of mortality did not convey all the information relevant to the prevention of tuberculosis mortality [20]. The PYLL metric subsequently achieved prominence in the burden of disease work of Murray and Lopez [21]. Currently, it is routine practice for the Australian Bureau of Statistics (ABS) [22] and the Australian Institute of Health and Welfare (AIHW) [23] to report both headcount and PYLL measures of suicide. A small number of Australian analyses have applied both headcount and PYLL measurement to suicide [24-26]. These studies show the added information gained by applying both headcount and PYLL metrics.

The focus here is on providing historical and comparative (with respect to other diseases/conditions) analyses through time, using headcount and PYLL measures. The measure of mental health-related mortality that we apply here involves summing across the relevant ICD codes through time both "suicide" and mortality from "mental and behavioural disorders". We take these combined causes of death to approximate the (mortality) size of the problem of mental health-related mortality.

The recent emphasis in burden of disease studies suggests that measuring morbidity as well as mortality is important. However, a limitation of the (Australian) burden of disease work is its cross-sectional nature; that is, data have been constructed for only two years, 1996 and 2003 [1,27]. This article examines 88 years of data.

The present study has a narrower focus, by measuring mortality in levels only; the focus does not extend to studying its distribution. Measuring the (age) distribution of mortality (due to all causes, or any cause) through time is possible, by applying the analytical framework pioneered by Silber [28-30] and Le Grand [31-33]; there is one Australian study which has measured the distribution of suicide [26]. Distribution topics in mental health-related mortality need further attention, but here the focus is on levels only.

There are several policy implications in applying the PYLL metric. The most important is that government policies usually are designed to affect a particular variable or target. When forming policy, or evaluating existing policy, such as evaluating the expenditure on Australia's National Suicide Prevention Strategy [34,35], it is important to use appropriate measures, as discussed elsewhere [24-26], of the variable being targeted. The PYLL metric provides relevant information for societal or policy issues, because it is a weighted measure (Table 1). This example shows clearly that the PYLL is a more appropriate measure of premature mortality, from a societal perspective, than the (equal) headcount measure.

**Table 1:** Two approaches to mortality measurement: the PYLL and the Headcount

Case	The PYLL metric	The count metric
(Given life expectancy US white women, 1940s, of 69 years)		
**Death of white woman, aged 24**	45 years of life	1 death
**Death of white woman, aged 62**	7 years of life	1 death

Source: Adapted from Dempsey[20]

Another policy implication relates to an argument from health economics. There has been considerable concern about the rising absolute and relative expenditures of health services [36-38]. For example, the focus from this perspective is that Australia's expenditure on health in 2004-05 was 8 per cent of Gross Domestic Product (GDP), whereas in 1960-61 it was 4.1 per cent of GDP [39]. The OECD average in 2003 was 8.8 per cent of GDP. Thus, comparatively, Australia's position is "in the middle", between the United States (15.0 per cent) and the United Kingdom (7.7 per cent). Even Australia's "middle" position is viewed with some concern, as other countries in a lower position must use their health resources differently from those above them [40]. Various economists, governments and others (e.g. insurance carriers) have adopted a cost-containment view. However, in the recent international literature on the economics of health services, the cost-containment emphasis has been subject to re-appraisal. This re-appraisal involves an examination of the contribution of the health sector in the totality of the economies of OECD-type countries. Scholars of the re-appraisal bring a different emphasis--it is argued that due regard must be given to the gains to health that both public health programs and clinical medicine have wrought [41-45]. In this context, accurate measurement of "the gains" is vital. The Discussion section below develops this point.

## Methods

In order to extract data on mental health-related mortality, annual data were summed across the relevant ICD codes for "suicide" and "mental/behavioural disorders", by five year age-groups, from the AIHW [23] and for the years prior to 1968, from ABS historical data [46-49]. The data set thus obtained provides a complete enumeration, though underestimated [50], of mental health-related mortality in Australia; it is not a sample. Given the various changes in the structure of, and particular codes in, the revisions of the ICD, we referred to Taylor's detailed accounts to guide the data recording [51]. Australian data on suicide start from 1907, but data on mortality from mental/behavioural disorders are available only from 1916. The data on All Causes, cancer and circulatory diseases are available from 1907. However, motor vehicle accident mortality data exist only from 1924.

The above data are of a headcount kind, and they are employed in the calculation of PYLLs. The ABS provides one account of the processes applied to headcount data in order to generate PYLL data [52]. The following equation conveys the method. It specifies the PYLL for Suicide for year *t*, and an assumed life expectancy of 75 years:

where Suicide PYLL(75)_AS _is the total PYLLs due to Suicide, age-standardised, at time period *t; D*_*s *_is the number of Suicides per age group; *A*_*s *_is the adjusted age at death due to Suicide per age group; and *i *is the number of age groups for *i *= 1, ... *n*.

In the present study, the 85+ age group is open in the raw data. We closed it at 100 years of age in our PYLL calculations. Also, we applied conventional age standardisation techniques, using the Australian Standard Population 1991, both to the headcount and the PYLL data sets.

Earlier in this Section the problem of under-estimation of suicide data, which is a well-documented problem, was raised. We have, elsewhere, quantitatively investigated one implication of this problem for PYLL measurement [25]. That very specific implication relates to the eighth and subsequent revisions of the ICD, which involved new codes for "deaths undetermined whether accidentally or purposely inflicted" (ICD codes E980-E989 in ICD-8 and ICD-9, and Y10-Y34 in ICD-10). We noted that non-inclusion of such deaths in that study [25], subsequent to 1968, could under-estimate the contribution of suicide, and thus overall mental-related mortality. Hence, we re-analysed the post-1967 data with these additional codes included. We found all measures increased due to this exercise, though the magnitude was very small both for headcounts and for PYLLs, because there were so few cases in the "undetermined" category. Hence, no substantive conclusions drawn in that paper were found to be affected by that under-reporting problem.

However, other problems exist in the accuracy of suicide data. It is very important to realise that our PYLL calculations are based on the mortality coding undertaken (and published) by the ABS. It has been known for some time that ABS data on suicide have been subject to misclassification [53], as well as other more general errors indicated by De Leo [40], De Leo, Klieve and Milner [54] and Walker, Madden and Chen [55]. These problems have become more important since 2000.

Harrison, Painter and Elnour [56] have undertaken an important study in which they re-worked the published ABS data for a single year, 2004. They found that the published ABS data for Australia underestimated suicide enumeration by 16 per cent. By far, the most important source of error was the non-inclusion of coroners' cases when they were incomplete at the time of ABS enumeration. (See also Elnour and Harrison [57].) The ABS for some years has published a "Caution" relating to the quality of published "cause of death" data and has announced changes to its processes of coding and publishing suicide data [58]. The Caution reads as follows: The level of recorded deaths attributed to suicide, and observed changes over time are likely to have been affected by delays in finalising a cause [59]. The ABS has, from 2009, commenced a process of revising suicide data as more information, such as coroners' decisions, becomes available.

However, the impact of this inaccuracy in the data for this study, and thus for PYLL calculations in general, is not known. This is because the age-distribution of that under-reporting is not known. Until known, the implication for PYLL measurement cannot be determined.

## Results

We report mortality rates (i.e. conventional rates based on headcount data) first.

Mortality rates: mental health-related and some other causes

**Figure 1:**
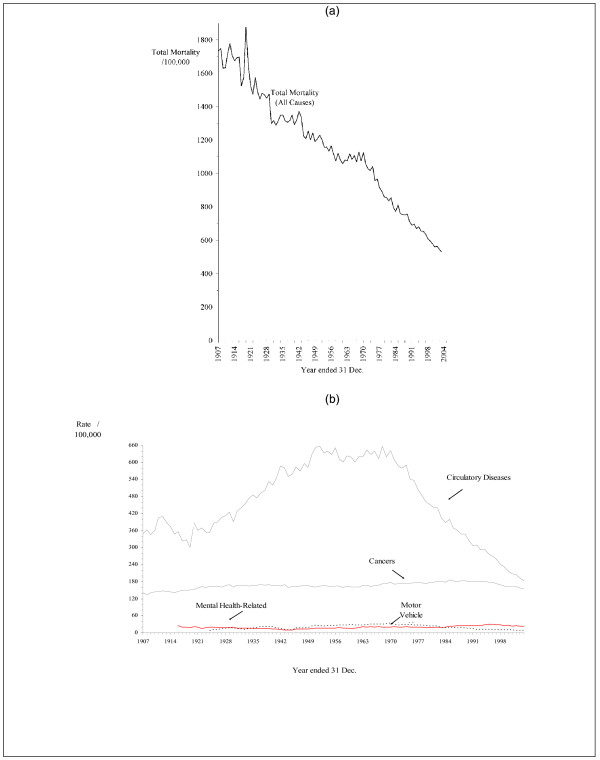
**Age-standardised mortality rates for (a) total mortality (all causes combined), and (b) circulatory diseases, cancers, mental health-related mortality and motor vehicle accidents, Australia, persons, 1907-2004**. * These rates have been standardised to the age distribution of the 1991 Australian population. ‡ Mental-Related Mortality includes Mental/Behavioural Disorders and Suicide. Sources: AIHW[23], CBCS [46-49], Taylor[52]

Figure 1 puts the mental health-related mortality trend in perspective by presenting a line graph of the All Causes mortality rate as well as comparative mortality rates associated with some causes of death, *viz*. mental health-related (as defined here), cancer, circulatory diseases and motor vehicle accidents. Given the large differences in age-standardised rates, the figure has two parts, Part (a) showing a long-run decrease in the All Causes mortality rate. This reflects the experience of many countries. Some argue that the nineteenth century witnessed a transition phase, and then a period of virtually continuous decline in mortality, and that this trend is an "epidemiological transition" or a "demographic transition" [60-63]. Part (b) depicts the mortality rates for four specific causes: circulatory diseases; cancers; motor vehicle accidents; and mental health-related.

Circulatory disease is by far the largest single cause of death and cancer is the second most important; mental health-related mortality and motor vehicle accidents are "small" by comparison. Note that there are differing temporal trends in these causes. The mortality rate for cancer has declined slightly over recent years. Mortality from circulatory diseases initially rose, but a large decline from 1968 has occurred. Mortality arising from motor vehicle accidents also was once rising, but it has declined after 1978. The fall in mortality due to both these causes has been substantial, but this is not the case with mental health-related mortality. The rising trend due to mental health-related mortality is noteworthy. It was relatively high in the 1920s, but it fell during the World War II period, and, since then, has risen, reaching a (local) maximum in 1996. The motor vehicle accident trend exceeded that for mental health-related mortality from the 1930s to the 1980s (and often by a substantial margin), but since 1983 the mortality rate from motor vehicle accidents is less than that for mental health-related mortality.

### Percentage contributions relative to All Causes

**Figure 2:**
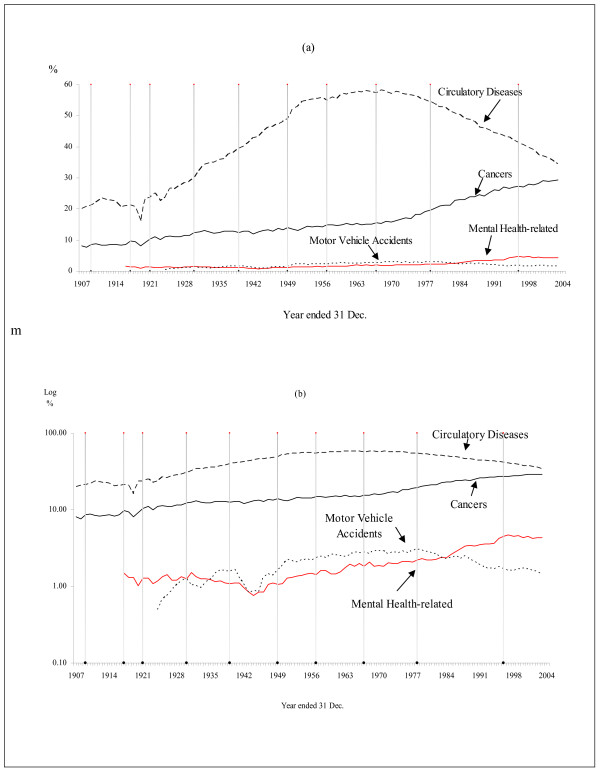
**Shares of five causes of death to all deaths, measured by counts, Australia, persons, 1907-2004**. The shaded vertical lines indicate the years of implementation of revisions of the ICD. Sources: AIHWAIHW[23], CBCS [46-49], Taylor[52]

Figure 2 depict the proportionate "shares" (or the percentage contributions) for each of these causes of death relative to All Causes through time. The calculation involved the ratio of each of the above four causes of death to All Causes, expressed as a percentage.

Figure 2 is also in two parts. With very large differences in the proportions for the particular causes presented here, the two-part depiction is helpful. The scale of the Y-axis in Part (a) is in levels, and is logarithmic in Part (b). The latter scale makes the detail about the temporal trends in the "smaller" causes of death clearer. Part (a) of Figure 2 shows that, during the 1950s-80s, circulatory diseases accounted for at least half the causes of death but that, since then, the relative fall in this cause of death is very marked, and accounted for 35 per cent of deaths in 2004. The relative importance of the contribution of cancer to all mortality has increased. Cancer accounted for approximately 10 per cent of all deaths in the 1940s, whereas the comparable figure was nearly 30 per cent of all deaths by 2004. Although the trends through time in the contributions of motor vehicle mortality rates and mental-health related mortality rates appear different from each other, the graphs in Part (a) are difficult to interpret. Part (b) clarifies the picture. In 1996, the relative importance of mental/behavioural disorders and suicide reached a maximum of nearly 5 per cent of all causes, and has remained at that approximate level in subsequent years.

While it is not our purpose to explain the trends depicted in Figures 1 and 2, it is useful to reflect on some known factors. The rise of motor transport early last century resulted in increased accident mortality through time, but governments eventually undertook many interventions, such as seat-belt legislation, crash helmets, safety-designed roads, drink-driving legislation etc. Some of these interventions are efficacious [64]. Similarly, for ischaemic heart disease and related conditions [65], medical treatments have improved and awareness has increased due to interventions undertaken by governments, particularly in the form of health information. While the data on mortality depicted here do not show the remarkable downturn in other causes of death, such as infectious diseases [66], the implementation of public health measures was successful in these areas [67]. In this context, note that the first year of the allocation of Australian Government funds to suicide prevention was 1995.

The message from both Figures 1 and 2 is clear: there is no evidence that the prevention of mental health-related mortality is working (as measured by the summation of deaths from mental/behavioural disorders and suicide). That is, no evidence is found that the demographic transition is presently operative for mental-health related mortality.

This conclusion is in line with those scholars who are concerned with analysing "avoidable mortality". Suicide and deaths from mental/behavioural disorders have not been included in any list of "avoidable death". We postpone further consideration of this point to the Discussion section below. We now report the results from applying a PYLL metric.

### The PYLL results

**Figure 3:**
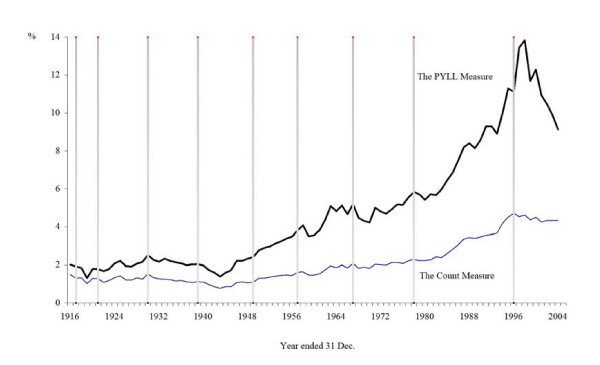
**Mental health-related mortality as a percentage of all causes measured by the count measure (no.) and the potential years of life lost (PYLL) measure, Australia, persons, 1916-2004**. Sources: AIHWAIHW[23], CBCS [46-49], Taylor[52]

Figure 3 as with Figure 2 is concerned with comparison. However, we now focus on mental health-related mortality, and compare the two measures of mortality (the headcount measure and the PYLL measure) of this aggregated cause of death. We have calculated the percentage shares of mental health-related mortality to All Causes mortality (measured by a headcount and by PYLLs). The percentage share for "The Headcount Measure" in Figure 3 is the same as the "Mental Health-Related" share in Figure 2, given that the measure of the rates of Figure 2 is the headcount.

It is clear that both measures of mental health-related mortality exhibit the same general patterns through time. Both measures show that the relative (numerical) importance of mental health-related mortality has risen since the end of World War II. However, the PYLL measure (which reaches a local maximum of nearly 14 per cent of All Causes mortality in 1998) indicates that the problems in the mental health sector are considerably larger than indicated by "The Count Measure" (nearly 5 per cent in 1998).

It may be thought that the apparently large downturn which can be observed in Figure 3 post-1998 in the PYLLs trend is of some importance, to the extent that the conclusions of the article are contradicted. In the following paragraphs we argue that any such position is not the case. First, we have elsewhere reported the results of estimating equations to time-series data, in an article that focuses on the distribution of suicide [26]. In that paper, where the reported equations are for rates, both on headcount data and on PYLL data, we find that the trend is not cubic: there is no downturn in those data. In other words, the Ramsey RESET test indicated that the post-1998 decrease was not statistically significant. Given that we subjected those equations to a full raft of diagnostic tests of the residuals, and the stability of the specification of the model, considerable confidence can be placed in those results.

Figure 3 is not a depiction of rates, but the trend in the contribution of mental health-related mortality to All Causes mortality. However, in the Methods section above, we explain that, for various reasons, the number of (published) suicides has been falling recently. Not even the ABS believes that "less suicide" is actually happening. Rather, what is happening is inaccuracies in the suicide data have been worsening recently. Recall that a re-working of the mortality data for 2004, the last year of our analysis here, indicated that the underestimation of the published data for that year amounted to 16 per cent. Furthermore, there is other evidence which indicates that the underestimation has been more severe since 2000 [56]. The data inaccuracy issue is addressed in more detail above, and Figure 3 reflects this problem. The PYLL measure particularly is accentuating the data inaccuracy of recent years.

Thus, the conclusions that one draws about the numerical importance of mental health-related mortality depends on the mortality measure employed. The headcount measure is the typical measure applied to the mental health sector: the results in Figure 3 clearly indicate that this measure of mortality underestimates the size of the problems associated with mental disorders in Australia. We undertook PYLL calculations for the contributions of the other three causes of death but we do not report these results here due to space limitations. These results are available from the authors on request.

Attention should be paid to both headcount and PYLL measures because each sheds light on different aspects of the phenomenon.

## Discussion

As argued briefly in the Background section of this article, we suggest that it is misplaced for policy makers to have a concern solely with health expenditure.

Attention should be directed to both health expenditure and the value of the health outputs produced by the health sector. A statement by Nordhaus neatly captures this perspective, as follows: "The new view of health economics should shape the way we think about health policy. In the early 1990s, the general hysteria about rising health costs led many to believe that the health care system was wasteful and out of control and should be reigned in" [p. 20] [68]. There is not the space to describe or review the reappraisal studies, but note that Davis et al. have argued cogently that this perspective is important, and that it does not negate the common criticisms levelled at the health sector, eg poor access, inappropriate treatment, issues arising with market power etc [69]. The point of the present article is made in this context: information about the relative numerical importance of mental disorders is very important.

These arguments suggest reflecting on the factors known already to contribute to the long-run decrease in mortality, which is characteristic of "the demographic transition". One key factor is knowledge of disease processes. For example, in nineteenth century England, the observational disposition of John Snow towards the water supply (wells etc.) ultimately provided the relevant knowledge of water contamination for the prevention of cholera; and knowledge of efficacious therapies, such as the Fleming-Florey "story" of the development of knowledge about antibiotics, is an example, of a different kind, of how knowledge is applied. A more recent factor is technological change. Technological change involves both life-saving technologies, such as organ transplantation, and "maintenance" types of technologies, such as dialysis for end-stage renal disease. Some technological change enhances the productivity of curative and preventive health services. This factor is far from trivial. Consider extra-corporeal shockwave lithotripsy, extracapsular cataract extraction and phaco-emulsification cataract therapy, which are three technologies of this kind.

Additionally, there are the technological changes that have occurred in diagnosis. For example, whereas once there was just radiology, there is now also MRI, multi-slice CT scanning, Dopler ultrasound, PET scanning, gamma camera imaging etc. The point of this paragraph is to emphasise the implication of this paper which is that an appropriate focus in research about the mental health sector is to determine, and implement, the factors that will contribute to the long-run decrease in mortality in the mental health sector.

We now qualify the argument here with four points. The first relates to the notion of "avoidable mortality" or "amenable mortality". Since Rutstein et al. [70], numerous scholars have formed lists of diseases/conditions for which medical or societal interventions are efficacious [71-74]. Nolte and McKee provide a comprehensive review [75]. It is noteworthy that suicide and mortality from mental/behavioural disorders are not included in any list of avoidable deaths: deaths from mental illnesses are classified as "unavoidable" in that literature. This classification can be confused with an implicit attitude of resistance towards allocating resources to averting suicide and all mental-related mortality, or passivity towards this cause of death, more so since it is classified as "unavoidable". The argument in this paper refutes any such stance.

The second point is technical, and relates to having relevant data available when studies to evaluate the efficacy of prevention strategies are sought. Given the suggestion that efficacious government policy can contribute to reversing the mortality trend from mental disorders, could such an impact be detected statistically? Bhattacharyya and Layton provide one example [64]. The task involves detecting (post-intervention) whether or not there has been a reversal in the sign on the slope variable of the equation for the mortality trend (i.e. from positive to negative). The above results also indicate the importance of observing the sign on the slope of all relevant trends: e.g. the age-standardised headcount rate, etc. Thus, the appropriate technique exists for establishing empirically the impact of a government policy on prevention of mental health-related mortality.

Third, as mentioned above, mortality is but a partial, and imperfect, measure of the health status of a community. Thus, this point is a qualification. Since Zeckhauser and Shepard [76] outlined the Quality Adjusted Life Year (QALY) concept, it is regular practice to consider the quality of life associated with morbidity, along with mortality. While the formation of time-series data sets of mental health-related morbidity is desirable, the "quality" of morbidity is a "gap" in health data-sets, and it is rarely discussed. The absence of such a time-series data set on morbidity has induced us to have recourse to mortality data: such data are available for a relatively long period. The results presented in this paper clearly indicate the importance of determining the shape and direction in the long-term trend in mortality from this specific cause of death. Determining even this much information is not trivial, even though the trend in the morbidity from mental conditions cannot be determined over that period.

Our final point is a qualification. The argument in our paper does not negate some other very important issues in the mental health sector. Such issues include unmet need in the provision of mental health services [77-79], and the matter of people with mental disorders not receiving efficacious, evidence-based treatments [80-82]. This paper is an exercise in descriptive science and it is not our purpose to take any normative stances or to enter the debates about these issues. Rather, we seek measurement approaches that will inform policy debates better.

## Conclusions

It is unbalanced to focus solely on rising health expenditures, without valuing the improvements in health status brought by those expenditures: it is important to consider health gains alongside expenditures. A re-thinking of the conventional wisdom about cost-containment does not imply that various criticisms of the provision of health services no longer apply, but it does suggest that determining the place of mental disorders in the context of the reappraisal literature is relevant. The above results are unambiguous: there is no evidence that long-run mental health-related mortality is falling (as measured here by the summation of deaths from mental/behavioural disorders and suicide) and that suicide and mental/behavioural mortality has not contributed to the decline in the All Causes mortality rate in Australia. The epidemiological transition is yet to come to the mental health sector.

Adequate levels of (public and private) mental health expenditure, when combined with efficacious mental health policy in Australia, will contribute to any lasting decline in the mental health-related illness or mortality trend. Finding services and treatments that "work" with mental disorders is important, as is applying them. Thus, there is a need for both basic and applied research on the causes of mental illness, and the development of efficacious therapies.

## Competing interests

The authors declare that they have no competing interests.

## Authors' contributions

DD and RW had the idea of conceptualising the issue considered here. This framework was outlined to HW, an early writer on measurement and data on mental health sector outcomes. All authors have made substantial contributions to conception and design, or to acquisition of data, or analysis and interpretation of data. They have all been involved in drafting the manuscript and revising it critically for important intellectual content. All three authors have given final approval of the version to be published. Each author has also participated sufficiently in this article to take public responsibility for the content of this article. Thus, all authors contributed to this paper.

None of our employers had any role in the conception and conduct of the study, the analysis of the data, or the first draft and subsequent revisions of the manuscript. All authors have reviewed and approved the final manuscript, and take responsibility for the content of the paper. The work reported here is independent of all our employers.
